# Identification of polymorphisms and balancing selection in the male infertility candidate gene, *ornithine decarboxylase antizyme 3*

**DOI:** 10.1186/1471-2350-7-27

**Published:** 2006-03-16

**Authors:** Greg L Christensen, Ivaylo P Ivanov, Stephen P Wooding, John F Atkins, Anna Mielnik, Peter N Schlegel, Douglas T Carrell

**Affiliations:** 1 Andrology and IVF Laboratories, University of Utah School of Medicine, Salt Lake City, UT, USA; 2 Department of Physiology, University of Utah School of Medicine, Salt Lake City, UT, USA; 3 Department of Human Genetics, University of Utah School of Medicine, Salt Lake City, UT, USA; 4 Biosciences Institute, University College, Cork, Ireland; 5 Department of Urology, Weil Medical College of Cornell University, New York, NY, USA; 6 Center for Biomedical Research, The Population Council, New York, NY, USA

## Abstract

**Background:**

The antizyme family is a group of small proteins that play a role in cell growth and division by regulating the biosynthesis of polyamines (putrescine, spermidine, spermine). Antizymes regulate polyamine levels primarily through binding ornithine decarboxylase (ODC), an enzyme key to polyamine production, and targeting ODC for destruction by the 26S proteosome. Ornithine decarboxylase antizyme 3 (OAZ3) is a testis-specific antizyme paralog and the only antizyme expressed in the mid to late stages of spermatogenesis.

**Methods:**

To see if mutations in the *OAZ3 *gene are responsible for some cases of male infertility, we sequenced and evaluated the genomic DNA of 192 infertile men, 48 men of known paternity, and 34 African aborigines from the Mbuti tribe in the Democratic Republic of the Congo. The coding sequence of *OAZ3 *was further screened for polymorphisms by SSCP analysis in the infertile group and an additional 250 general population controls. Identified polymorphisms in the *OAZ3 *gene were further subjected to a haplotype analysis using PHASE 2.02 and Arlequin 2.0 software programs.

**Results:**

A total of 23 polymorphisms were identified in the promoter, exons or intronic regions of *OAZ3*. The majority of these fell within a region of less than two kilobases. Two of the polymorphisms, -239 A/G in the promoter and 4280 C/T, a missense polymorphism in exon 5, may show evidence of association with male infertility. Haplotype analysis identified 15 different haplotypes, which can be separated into two divergent clusters.

**Conclusion:**

Mutations in the *OAZ3 *gene are not a common cause of male infertility. However, the presence of the two divergent haplotypes at high frequencies in all three of our subsamples (infertile, control, African) suggests that they have been maintained in the genome by balancing selection, which was supported by a test of Tajima's D statistic. Evidence for natural selection in this region implies that these haplotypes may be associated with a trait other than infertility. This trait may be related to another function of OAZ3 or a region in tight linkage disequilibrium to the gene.

## Background

Polyamines are small, ubiquitous organic molecules derived from arginine or methionine. They carry a positive charge on each nitrogen atom, which allows them to interact with polyanionic macromolecules like DNA, RNA and some proteins [1]. Often, these interactions are very specific, and play critical roles in normal cell growth and function [2]. Abnormal expression of polyamines has been associated with both apoptosis and tumor growth [3]. Consequently, polyamine concentrations are tightly regulated at transcriptional, translational, and post-translational levels [4].

Ornithine decarboxylase (ODC), which converts ornithine to the first polyamine, putrescine, is the rate-limiting step in polyamine biosynthesis and the key point at which polyamine levels are regulated [5]. In eukaryotic cells, the most important regulator of ODC is the protein ornithine decarboxylase antizyme (antizyme) [6]. When polyamine levels rise to a critical level they induce a +1 ribosomal frameshift in antizyme mRNA, producing a functional antizyme protein [7]. The antizyme protein binds ODC, preventing formation of the enzymatically-active ODC homodimer and targeting ODC for ubiquitin-independent proteolysis by the 26S proteosome [6]. In addition to inhibiting the production of intracellular polyamines, antizyme can also inhibit the import of extracellular polyamines [8].

Antizyme genes have been identified from yeast to mammals. Most invertebrates have a single antizyme gene, while there are at least three independently conserved antizyme isoforms in vertebrates [9]. Three main antizymes have been described in mammals. Antizyme 1 (AZ1) and antizyme 2 (AZ2) are generally thought to be present in all tissues except haploid male germ cells, with AZ1 mRNA being present in a 10- to 20- fold excess of AZ2 [10]. AZ1 is more active in the degradation of ODC than AZ2, and both contribute to the negative regulation of polyamine transport [1].

Antizyme 3 (OAZ3) is expressed predominanty in round and elongating spermatids. Though AZ1 is expressed in early, diploid testicular germ cells, OAZ3 is the only antizyme present in post-meiotic, male germ cells [11-13]. Expression of OAZ3 follows closely behind ODC expression, which is tightly regulated in the testis, with a sharp spike in expression occurring during the late spermatocyte and early spermatid stages [12]. Transgenic male mice that overexpress ODC, resulting in increased synthesis of polyamines are infertile, with an almost complete lack of mature spermatozoa [14,15] This suggests that OAZ3 might play an important role in maintaining spermatogenesis by controlling ODC and polyamine levels. A recent report indicates that OAZ3 may also interact with at least one additional protein in the testis, gametogenetin 1, [16], in an unspecified manner. The function of gametogenetin 1 is unknown, but its testis-specific expression is similar to OAZ3 both spatially and temporally.

Infertility affects approximately 10% of couples, and in about half of these cases a male factor problem can be identified [17]. The etiology of male factor infertility is poorly understood. While some can be explained due to Y-chromosome microdeletions, endocrine disruptions, developmental abnormalities or environmental insults, the majority remains idiopathic and potentially genetic in origin [18]. Multiple studies, primarily through transgenic animal models, have helped develop a list of candidate genes that may contribute to human, male infertility [19,20]. Given its testis-specific expression and the clear link between deregulation of polyamine expression and infertility [21], *OAZ3 *is a clear choice for a male infertility candidate gene.

To determine if mutations in *OAZ3 *account for some cases of male infertility we screened 192 patients and 334 fertile or general population controls by either single-strand conformational polymorphism analysis (SSCP) or directly sequencing the exons and flanking intronic sequence of *OAZ3*. In addition to screening the *OAZ3 *gene for possible mutations associated with infertility we have also examined the frequency of haplotypes that emerged and the evolutionary relationships among the haplotypes. Finally, to examine the relationship between variation in this gene and disease phenotype, we tested for associations between haplotype and phenotype.

## Methods

### Patient and control DNA samples

After obtaining Institutional Review Board approval, a total of 192 men presenting with primary infertility were enrolled at the University of Utah School of Medicine (Salt Lake City, UT) or the Weill-Cornell Medical Center (New York, NY). The patients were either azoospermic, having no sperm in the ejaculate (n = 143) or severely oligozoospermic, having less than five million sperm/ml. of semen (n = 49). These patients were selected because their phenotypes match those observed in mice overexpressing ODC, which exhibit both azoospermia, primarily due to maturation arrest, and oligozoospermia [14,15]. To increase the chance of identifying a mutation in the *OAZ3 *gene potentially responsible for male infertility or subfertility, and to decrease possible confounding factors, patients were excluded if they had any suspected or known causes of male infertility. For example, patients with a known Y-chromosome microdeletion, cystic fibrosis, varicocele, Klinefelter's, exposure to chemotherapeutics or radiation etc., were not included. Venous blood was drawn from each patient using standard techniques and DNA extracted using the Puregene DNA extraction kit (Genzyme, Minneapolis, MN).

In addition to the infertile group, all or part of *OAZ3 *was screened in three different control groups. The first group (n = 48), representing men with established paternity, was obtained from the Utah Genetic Reference Project (UGRP). These individuals are primarily of Northern European ancestry and are genetically very similar to the infertile group. The second group was a sample of central African aborigines (n = 34), all members of the Mbuti tribe from the Democratic Republic of the Congo. The group is predominantly male, though there are a few females in the group. It is unknown which, if any of the group, are infertile. They were included in the study because they represent a group of genetically diverse individuals, geographically isolated from the infertile and fertile study groups, which is useful in determining how haplotypes may have evolved. The infertile and African groups were screened identically to the patient group by direct sequencing. A third and final general population group of men (n = 250), which included 90 African and 80 Asian males, was screened by SSCP analysis, specifically for the Pro164Ser change identified in one of the patient samples, but also for mutations in the coding regions in the rest of the gene. To assess evolutionary relationships with non-human primates, DNA was obtained and sequenced for both the common chimpanzee (*Pan troglodytes*) and gorilla (*Gorilla gorilla*).

### Mutation screening

Primers were designed to amplify the promoter and five coding exons of *OAZ3 *with its flanking intronic sequence, using standard polymerase chain reaction (PCR) techniques (Table 1). Primers were designed based on the OAZ3 sequence contained in genomic contig NT_004487. Thermocycling conditions were as follows: 94°C for 4 minutes followed by 35 cycles of 94°C for 30 seconds, annealing temperature of 60°C for 30 seconds, base extension 1 minute per kb at 72°C, and a final hold for 5 minutes at 72°C. Primary PCR products were cleaned-up using a gaunidium HCl protocol and sequenced in the forward and reverse directions on an ABI 3700 capillary sequencer.

**Table 1:** PCR and sequencing primers for the *OAZ3 *gene. An "F" in the name designates a forward primer, an "R" designates a reverse primer. The position given for each primer is that of the first nucleotide, in relation to the first nucleotide of the ATG start codon.

Region				
**Primer positions**				
PCR size	Name	PCR Primers 5'-3'	Name, **Position**	Sequencing Primers 5'-3'
Promoter	F-P	CTAATCAGGTCACCACTGGATCAGAGCC	SF-P1, **-518**	ACTGGATCAGAGCC
**-533, 16**	R-P	AACAACGAGGCAGCATCTTC	SF-P2, **-283**	GGAGTCCTGAGGTGA
549 bp	SR-P1, **16**	AACAACGAGGCAGCATCTTC		

EXON 1–3	F-1	CTACCTCTACCCGATCTGGTCACCA	SF-131, **-64**	GATCTGGTCACCATACGCC
**-76, 1446**	R-1	CCTTATGTACTATGACATGATAGAAGGG	SF-132, **231**	CCTGGTACTGTGTATCTTCCCACCTC
1522 bp			SF-133, **788**	CCTGGTTGTAACCATGGCA
			SR-131, **557**	CACATGCGTTCCAAGATTCCATCTACC
			SR-132, **1446**	CCCCTACCTATTCCCTCCCCATTC

EXON 4–5, 3'UTR	F-4	CAATCCCGGCACCTC	SF-451, **2978**	CTGGCAGATCACTCGTGGTTAG
**2951, 4841**	R-4	GAATGCCCTCTTCTA	SF-452, **3216**	GAGGGAGAGGGAGCGGCCAAACA
1890 bp			SF-453, **3831**	GAATAACCATTCATAAGTGA
			SF-454, **3425**	GCTGAGGCAGCTGGATAACT
*(PCR fragment includes 487 bp of the 3'UTR)*			SR-451, **3443**	GTTATCCAGCTGCCTCAGCGCG
			SR-452, **3966**	CCTCACAGAATTTAAGA
			SR-453, **4399**	ATGTGTCAAGGCTTCCAGCCCCTC
			SR-454, **4841**	GAATGCCCTCTTCTA

Sequence trace files generated from the ABI 3700s were analyzed using the Phred, Phrap, and Consed software programs [22]. Phred assigns a quantitative value to the quality of each sequenced base. This base quality provides a probabilistic estimate of the correctness of the base call. The sequences were assembled, and a consensus sequence generated from the most common base calls using the Phrap program, and potential mutations identified using Consed, which has the ability to search for high quality base discrepancies, based on the Phred values, in the assembled sequence. A visual analysis was also conducted of the trace files to confirm identified polymorphisms and potential mutations. In addition to direct sequencing, the five coding exons of the 192 patient samples and exon 5 of the 250-member panel were screened for variants by SSCP. This was done according to methods described previously [23].

### Haplotype analysis

Haplotypes were inferred using PHASE 2.02 [24]. Evolutionary relationships among haplotypes were inferred using Arlequin 2.0 [25] to generate minimum spanning trees. Population genetic diversity was measured using two standard statistics, S and pi. S is the number of variable nucleotide positions in a sample, pi is the mean pairwise difference (per nucleotide) between sampled sequences. Pairwise differences among sequences were calculated by counting the number of nucleotide positions distinguishing each possible combination of the 2*N sequences in each sample, where N is the number of individuals in the sample. Tests of evolutionary neutrality were performed using Tajima's D statistic [26]. Tajima's D compares S and pi to identify departures from neutrality and constant population size. Because much evidence suggests that modern human populations are the product of a major population expansion in the Upper Pleistocene [27-29], we took population size change into account in D tests using the method of Wooding et al. [30].

## Results

### Mutational analysis of *OAZ3*

**Figure 1:**
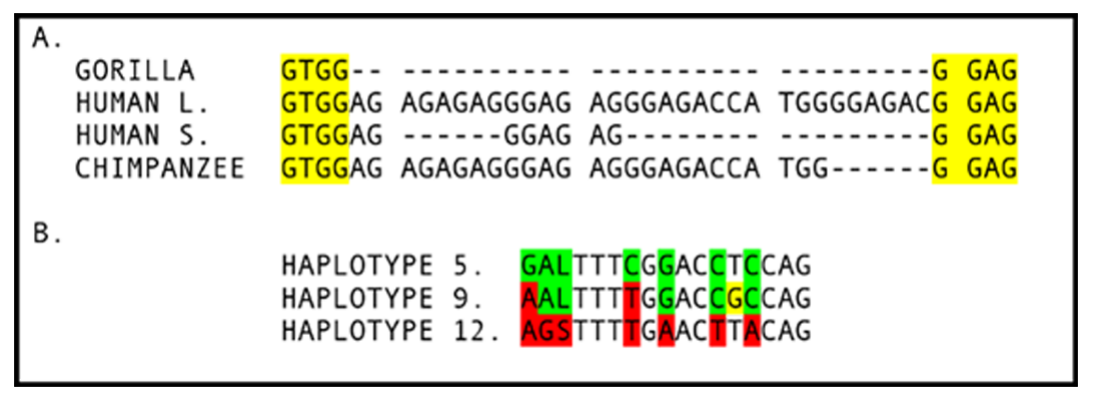
Alignment of nucleotide variant 8 (Table 1) and major haplotypes. A) Variant 8, located in intron 3 of *OAZ3 *was present in the long form (HUMAN L) in haplotype 5, and the short form (HUMAN S) in haplotype 12. Gorilla and chimpanzee sequences shown for reference. B) Alignment of the three major haplotypes, 5, 9, and 12. The position of the long or short allele shown in A is inserted as an "L" or "S" at the appropriate position. The positions unique to haplotypes 5 and 12 are colored in green or red, respectively. The figure depicts how the third major haplotype, 9, may have originated from both gene conversion and recombination between haplotypes 5 and 12. The alternate nucleotides present in each haplotype are identified in Figure 3.

To examine the role of OAZ3 in male infertility, genomic DNA was obtained from 192 infertile men, 143 with nonobstructive azoospermia and 49 with severe oligospermia (< 5 million sperm/ml). An additional 48 subjects of known fertility and 34 central African aborigines were also included as controls. The entire coding sequence, promoter region, and some intronic regions were analyzed for sequence variations by direct sequencing. The coding sequence of *OAZ3 *was also screened by SSCP in the infertile patients. A total of 23 variations were found (Table 2). Twenty of the variants (1–5, 7, 9–22) were single nucleotide substitutions, and two (6, 23) were deletions of a single nucleotide. The final variant (8), located in intron 3, occurred as either a 31 base pair or 8 base pair alternative (Figure 1) in all but one fertile patient, who had a heterozygous deletion of variant 8.

**Table 2:** Nucleotide variants detected in the human *OAZ3 *gene

Variant Number	Region	Nucleotide change	Amino acid change	NCBI	Allele frequency Infertile		Allele frequency Fertile			Allele frequency African	
1	Promoter	-239 A – G	-	NEW	[A] .997	[G] .003	[A] 1	[G] 0		-	
2	Intron 2	1079 A – G	-	rs12066445	[A] .66	[G] .34	-			-	
3	Intron 3	2992 G – C*	-	rs11204884	[G] .99	[C] .01	-			-	
4	Intron 3	3043 A – G	-	NEW	[A] .99	[G] .01	-			-	
5	Intron 3	3068 G – A	-	rs6667249	[G] .35	[A] .65	[G] .33	[A] .67		[G] .23	[A] .77
6	Intron 3	3079 C – 0	-	NEW	[C] .65	[DEL] .35	[C] .67	[DEL] .33		[C] .81	[DEL] .19
7	Intron 3	3153 A – G	-	RS4995159	[A] .51	[G] .49	[A] .48	[G] .52		[A] .81	[G] .19
8	Intron 3	See Figure 1	-	NEW**	[Long] .51	[Short] .49	[Long] .49	[Short] .5	[DEL] .01	[Long] .76	[Short] .24
9	Intron 3	3280 T – A	-	NEW	[T] .997	[A] .003	[T] 1	[A] 0		[T] 1	[A] 0
10	Intron 4	3420 T – G	-	NEW	[T] .994	[G] .006	[T] 1	[G] 0		[T] .94	[G] .06
11	Intron 4	3427 T – G	-	NEW	[T] .997	[G] .003	[T] 1	[G] 0		[T] 1	[G] 0
12	Intron 4	3457 T – C	-	rs3748612	[T] .65	[C] .35	[T] .65	[C] .35		[T] .86	[C] .14
13	Intron 4	3595 G – T	-	NEW	[G] 1	[T] 0	[G] 1	[T] 0		[G] .96	[T] .04
14	Intron 4	3619 G – A	-	rs1781424	[G] .52	[A] .48	[G] .49	[A] .51		[G] .75	[A] .25
15	Intron 4	3710 A – G	-	NEW	[A] 1	[G] 0	[A] 1	[G] 0		[A] .98	[G] .02
16	Intron 4	3735 C – T	-	NEW	[C] 1	[T] 0	[C] 1	[T] 0		[C] .98	[T] .02
17	Intron 4	3802 C – T	-	rs1781423	[C] .52	[T] .48	[C] .49	[T] .51		[C] .77	[T] .23
18	Intron 4	3934 T – G	-	rs6673002	[T] .91	[G] .09	[T] .88	[G] .12		[T] .61	[G] .39
19	Intron 4	4023 C – A	-	rs1781420	[C] .52	[A] .48	[C] .49	[A] .51		[C] .75	[A] .25
20	Exon 5	4280 C – T	P164S	NEW	[C] .997	[T] .003	[C] 1	[T] 0		[C] 1	[T] 0
21	3' UTR	4394 A – C	-	NEW	[A] 1	[C] 0	[A] 1	[C] 0		[A] .95	[C] .05
22	3' UTR	4487 G – C	-	NEW	[G] .99	[C] .01	[G] 1	[C] 0		[G] 1	[C] 0
23	3' UTR	4531 A – 0	-	rs3833528	[A] .52	[DEL] .48	[A] .49	[DEL] .51		[A] .72	[DEL] .28

* NCBI database shows a G/A SNP

** NCBI shows two SNPs within the alternate region, rs4995158, rs12750154

Ten of the identified variants (2, 3, 5, 7, 12, 14, 17–19, 23) (Table 2) had been reported previously in dbSNPs [31]. Five of the remaining 13 nucleotide variants, numbers 1, 9, 11, 20, and 22 were unique to the infertile group while four variants, numbers 13, 15, 16 and 21 were unique to the aborigines. No significant differences were observed in allele frequencies between the infertile and control group. Significant differences in allele frequencies were found between the African group and both of the other two groups for all polymorphisms identified that were present in more than one individual (p < .001).

Assuming OAZ3 plays an integral role in spermatogenesis, At least two of the identified variants, variants 1 and 20, may be candidates for involvement in male infertility or subfertility. Variant 1, -239A→G, identified in a heterozygous state in the 5' untranslated region (5'UTR) of an azoospermic patient, generates a new potential translation initiation ATG codon within a Kozak consensus sequence and occurs two basepairs downstream from a putative Inr binding site. Though translation initiated at this position encounters a stop codon just prior to the normal translation initiation site, it could potentially reduce the amount of OAZ3 mRNA available to translate. In this way it may reduce the total amount of OAZ3 in the testis and affect fertility.

**Figure 2:**
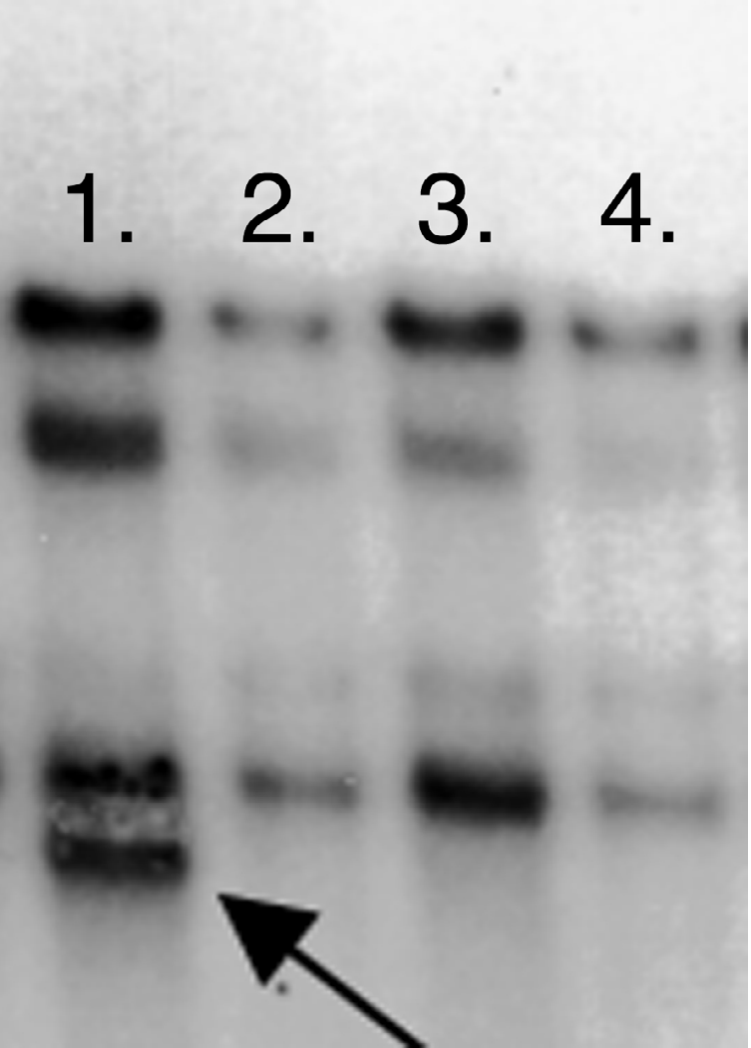
Segregation of SSCP bands for exon 5 of *OAZ3*. The arrow identifies the band representing the C→T, Pro164Ser polymorphism in a single azoospermic patient. Lanes 2–4 are infertile patients with the C nucleotide. All SSCP results were further confirmed by direct sequencing.

The second variant, variant 20 (4280C→T) in exon 5, results in the amino acid change Pro164Ser, and was identified in the heterozygous state in a single azoospermic patient by both direct sequencing and SSCP (Figure 2). This proline residue is conserved in human, mouse, rat, cow, pig, dog and opossum OAZ3. To further evaluate the significance of this change, exon 5 was screened by SSCP in an additional 250 controls, including 90 African and 80 Asian males. The P164S variant was not identified in this group, or any of the other controls. The patient was also negative for microdeletions of the Y-chromosome, the most common genetic cause of male infertility, using the Promega Y-chromosome microdeletion kit (Madison, WI). The parents and family of the affected individual were not available for screening to explore the possibility of haploinsufficiency produced by the mutation.

### Haplotype analysis

Of the 23 variants identified by direct sequencing, 16 were used for haplotype analysis. The 7 variants not used consisted of the 3 insertion/deletion polymorphisms (6, 8, 23) and 4 variants (1–4) for which complete data was not available in all samples. Of the 16 variable nucleotides used, separate analyses of the affected, control, and African subsamples revealed 12, 7, and 12 variable nucleotide positions, respectively.

**Figure 3:**
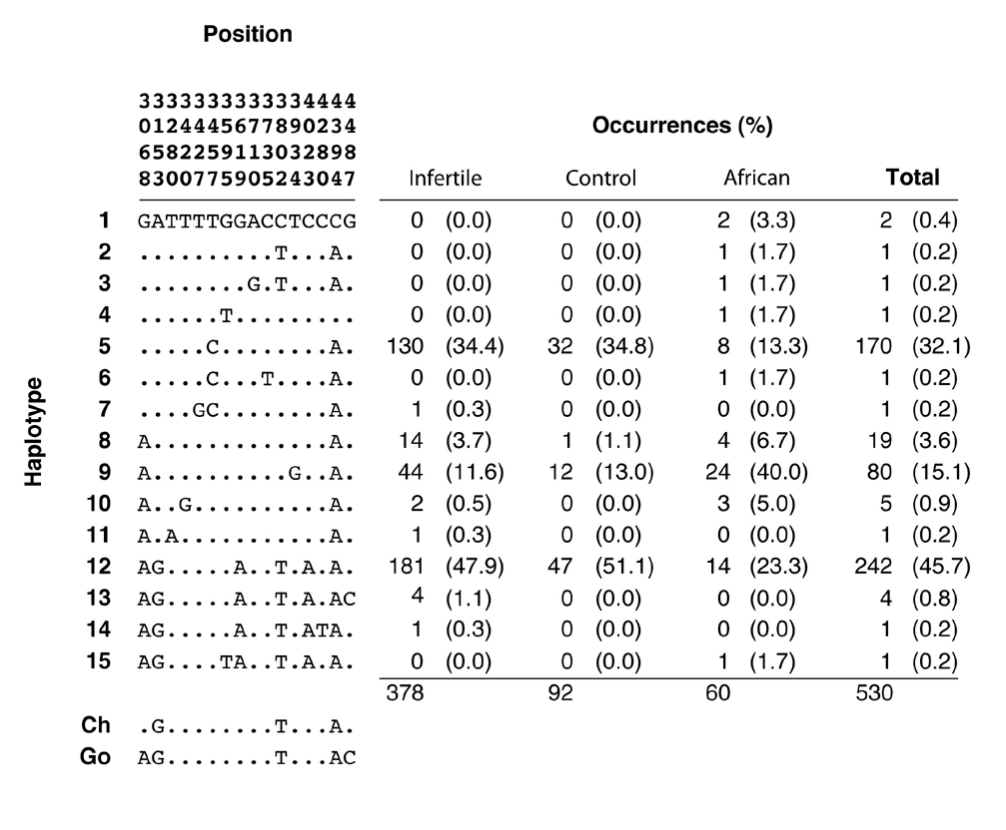
Summary of inferred haplotype data. In the left panel, each column represents a variable nucleotide position and the row indicates the nucleotide found at that position in the given haplotype. Dots indicate identity with the reference haplotype (arbitrarily chosen from among the haplotypes inferred in our sample). In the right panel, numbers indicate the number of occurrences of each haplotype in each subsample. Ch = chimpanzee, Go = gorilla.

Analysis using the PHASE computer program indicated that the nucleotide variants were partitioned into 15 haplotypes (Figure 3). Most haplotypes were observed just once or twice in the sample, and haplotype frequencies were similar among the affected, control, and African subsamples. Three haplotypes (5, 9, and 12) accounted for more than 90% of observations and these were the most common haplotypes in all subsamples. However, while haplotype 12 was the most common haplotype in the affected and control subpopulations, followed by haplotype 5 and then haplotype 9, haplotype 9 was the most common haplotype in the African subsample.

When haplotypes 5, 9, and 12 were aligned, it was apparent that haplotype 9 was derived from haplotypes 5 and 12. This appears to have taken place as the result of a gene conversion event coupled with a recombination event (Figure 1). No intermediary haplotypes between 5 and 9 or 12 and 9 were detected, suggesting that the gene conversion and recombination event from which 9 was derived must have occurred very close together historically.

**Figure 4:**
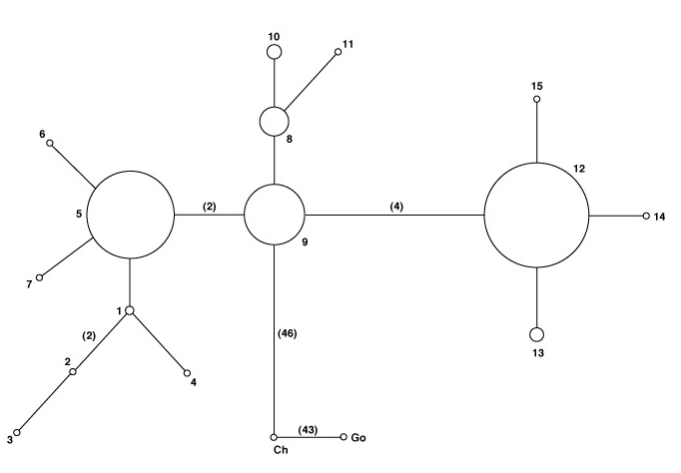
Minimum spanning tree relating inferred haplotypes. Each numbered node (circle) represents a haplotype, and the area of the node represents the haplotype's frequency. Edges (lines between nodes) represent nucleotide differences. Each edge represents one nucleotide difference unless indicated otherwise in parentheses. Ch = chimpanzee, Go = gorilla.

The two most common haplotypes in the sample, 5 and 12 (which together accounted for more than 75% of all observations) differed at six nucleotide positions (Figure 4). For this reason, the mean pairwise difference among sequences was relatively high: 3.2nt in the sample as a whole and 3.1nt, 3.2nt, and 3.0nt in the infertile, control, and African subsamples, respectively.

**Figure 5:**
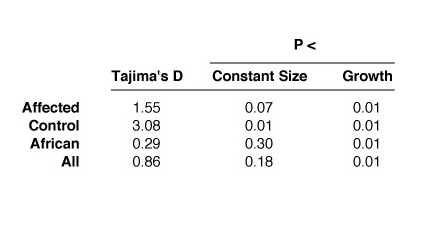
Results of Tajima's D test. The column labeled "Constant Size" shows the one-sided p-value of Tajima's D under the assumption that human population size has been constant. The column labeled "Growth" shows the one-sided p-value of D under the more realistic assumption that the human population expanded 100-fold, 100,000 years ago.

Tests of evolutionary neutrality were performed using Tajima's D statistic (Figure 5), which is conservative in the presence of recombination, revealed that D did not depart significantly from neutral expectation under the assumption of constant population size except in the control population. However, under the more realistic assumption that the human population expanded 100-fold from an initial effective size of 10,000, 100,000 years ago, the hypothesis of neutrality was rejected in all cases with p < 0.01.

## Discussion

The population of infertile patients we screened displayed phenotypes consistent with what we expected to observe if they harbored debilitating mutations in *OAZ3*. Our results indicate that mutations in the *OAZ3 *gene are not strongly associated with human male infertility, though they may contribute to infertility in isolated instances. It is also possible that mutations in *OAZ3 *result in a phenotype different than the one we selected for. If that is the case, information from the OAZ3 knockout mouse, currently in progress, may provide a clue as to which human phenotype should be screened. One patient had a heterozygous change of a single, nonpolar proline residue to a charged, polar serine residue in exon five, Pro164Ser. It is not known if the proline residue is part of a functional domain or how its alteration might affect the function of the OAZ3 protein. It is conserved in all of the other OAZ3 sequences examined (Gorilla, chimpanzee, rat, mouse). We are aware of at least four proteins that interact with OAZ3 (unpublished data) and the proline residue may be important for correct protein interaction. The variation was not identified in any of the additional 191 infertile or 332 control individuals screened, indicating it is very rare. Currently, there is no direct evidence that the Pro164Ser mutation affects OAZ3 function. Additional studies will need to be conducted to determine how this mutation affects OAZ3 and spermatogenesis. Variant 1, a -239A→G change in the 5' UTR of one infertile patient may also be of significance, as it was not identified in the fertile control group and might lead to reduced levels of OAZ3 being translated. The -239A→G variant also occurs two basepairs downstream from a potential Inr transcription factor binding site [13], which may possibly attenuate the transcription factor's interaction with the binding sequence.

Based on the NCBI SNPs database, at least 10 additional SNPs, which we did not identify, are located in the region we screened. Only one of these, rs 5777784, appears to have potential clinical significance. It shows the deletion of a guanidine in exon three, which would cause a frameshift mutation in *OAZ3 *and a truncated protein. As we did not detect this SNP in any of our samples, we cannot verify its existence.

The presence of two divergent haplotype clusters in our sample suggested that either balancing natural selection or population subdivision has been active in our study population. Both of these factors can maintain divergent alleles for extended periods of time [32]. Balancing natural selection, for example, can maintain divergent alleles when heterozygotes have a fitness advantage, while population subdivision can maintain divergent alleles in different populations. However, the presence of the two divergent haplotypes at high frequencies in all three of our subsamples (infertile, control, and African) shows that subdivision cannot account for the presence of these divergent lineages. A better explanation is that balancing natural selection has historically been active in this region of the genome, or in some region in tight linkage disequilibrium with it.

To test whether balancing selection might be occurring in a region connected to *OAZ3*, we reviewed SNPs identified in *OAZ3*s nearest neighbors that might have functional implications for the protein. Only one SNP, an A→G that converts a histidine residue to arginine in the final exon of the tudor and KH domain containing (*TDRKH*) gene, was identified. The 3' end of *TDRKH*, located on the opposite strand of *OAZ3*, overlaps with the 3' end of *OAZ3*, and the SNP in question lies in close proximity to the region from which the haplotypes were derived. The region containing the *TDRKH *SNP was amplified by PCR in 12 samples, six from each of the two major haplotypes and sequenced. While the SNP was identified in several of the patients, it appeared in both major haplotypes, indicating a different allele in the region is the one being maintained by balancing selection.

Evidence that balancing natural selection may have been active in the *OAZ3 *region is further supported by tests of Tajima's D statistic [26]. Tajima's D compares estimates of diversity based on S and pi, which are affected differently by natural selection. Positive natural selection, for instance, which drives a new favored variant rapidly to high frequency, tends to reduce pi relative to S, causing negative values of D. In contrast, balancing natural selection tends to increase pi relative to S, causing positive values of D.

Initial tests of Tajima's D statistic showed that the D values observed in our sample, while positive, were not significantly different from neutral expectation (Figure 5). However, these initial tests were performed under the assumption that human population sizes have been constant. This assumption is likely inappropriate, because archaeological and genetic evidence suggest that human populations expanded greatly – more than 100-fold – beginning roughly 100,000 years ago [33,34]. For this reason, we retested Tajima's D statistic under the conservative assumption that the human population increased 100-fold, from an initial effective population size of 10,000 (a typical estimate; [28]), 100,000 years ago. Under this assumption, the observed values of D departed from expectation in every subsample, and in the sample as a whole. Thus, patterns of variation in this region are consistent with the hypothesis that balancing natural selection has been active.

One possible explanation for balancing selection is heterozygote advantage, which can result from increased homozygote mortality or increased heterozygote reproductive success. To test for this possibility we performed Chi-squared tests of Hardy-Weinberg equilibrium on each SNP in our sample. Only two variants, variant 18 in the infertile group and variant 7 in the African group were significantly different than expected (p < .01). These tests show no evidence of a heterozygote excess and suggest that balancing selection in *OAZ3 *would be better explained by differences in pure reproductive success than by differences in survivorship. In the case of favored reproductive success, no excess of heterozygotes is expected, as random matings will return the population to Hardy-Weinberg equilibrium.

Signatures of natural selection can only arise when some sort of variation with phenotypic consequences, upon which selection can act, is present. Thus, evidence that balancing natural selection has been active in *OAZ3 *suggests that some kind of functional variation is present in or near this region. This variation is likely in linkage disequilibrium with the genetic variants that distinguish the two major haplotypes, which seem to have been maintained by selection. While the similar frequencies of these divergent haplotypes in our affected and control samples suggests that they are not likely associated with the phenotype examined here, they could be associated with other phenotypes related to OAZ3.

## Conclusion

Polymorphisms in *OAZ3 *are not strongly associated with human male infertility, though some of the identified SNPs, like Pro164Ser in exon 5, may contribute to isolated cases of infertility. The presence of two divergent haplotype clusters in *OAZ3*, in all subsamples tested (infertile, control, African), implies the haplotypes are being maintained by balancing natural selection. Natural selection in this area indicates these haplotypes may be associated with a trait other than infertility. This trait may be related to either a different function of OAZ3 or a region in tight linkage disequilibrium to the *OAZ3 *gene.

## Competing interests

The author(s) declare that they have no competing interests.

## Authors' contributions

GC collected patient study samples, participated in experiments, collected and analyzed data, and drafted the manuscript. II contributed to data collection, analysis of sequencing reads, supervision of the study and revision of the manuscript. SW contributed to the haplotype analysis and writing of the manuscript. JA participated in outlining the study, interpretation of results, and manuscript revision. AM assisted in collection and preparation of study samples. PS contributed to sample collection and revision of the manuscript. DC assisted in outlining the study design, directing research and revising the manuscript.

## Pre-publication history

The pre-publication history for this paper can be accessed here:


